# Cyber and Traditional Bullying Victimization as a Risk Factor for Mental Health Problems and Suicidal Ideation in Adolescents

**DOI:** 10.1371/journal.pone.0094026

**Published:** 2014-04-09

**Authors:** Rienke Bannink, Suzanne Broeren, Petra M. van de Looij – Jansen, Frouwkje G. de Waart, Hein Raat

**Affiliations:** 1 Department of Public Health, Erasmus University Medical Center Rotterdam, Rotterdam, the Netherlands; 2 Municipal Public Health Service Rotterdam area, Rotterdam, the Netherlands; University of Rochester, United States of America

## Abstract

**Purpose:**

To examine whether traditional and cyber bullying victimization were associated with adolescent's mental health problems and suicidal ideation at two-year follow-up. Gender differences were explored to determine whether bullying affects boys and girls differently.

**Methods:**

A two-year longitudinal study was conducted among first-year secondary school students *(N* = 3181). Traditional and cyber bullying victimization were assessed at baseline, whereas mental health status and suicidal ideation were assessed at baseline and follow-up by means of self-report questionnaires. Logistic regression analyses were conducted to assess associations between these variables while controlling for baseline problems. Additionally, we tested whether gender differences in mental health and suicidal ideation were present for the two types of bullying.

**Results:**

There was a significant interaction between gender and traditional bullying victimization and between gender and cyber bullying victimization on mental health problems. Among boys, traditional and cyber bullying victimization were not related to mental health problems after controlling for baseline mental health. Among girls, both traditional and cyber bullying victimization were associated with mental health problems after controlling for baseline mental health. No significant interaction between gender and traditional or cyber bullying victimization on suicidal ideation was found. Traditional bullying victimization was associated with suicidal ideation, whereas cyber bullying victimization was not associated with suicidal ideation after controlling for baseline suicidal ideation.

**Conclusions:**

Traditional bullying victimization is associated with an increased risk of suicidal ideation, whereas traditional, as well as cyber bullying victimization is associated with an increased risk of mental health problems among girls. These findings stress the importance of programs aimed at reducing bullying behavior, especially because early-onset mental health problems may pose a risk for the development of psychiatric disorders in adulthood.

## Introduction

Recent studies indicate that approximately 20–35% of adolescents report involvement in traditional, offline bullying either as a bully, a victim or both [1]. Bullying can be defined as an aggressive act that is carried out by a group or an individual repeatedly and over time against a victim who cannot easily defend himself or herself [2]. Traditionally, four main types of bullying are distinguished: physical (e.g., assault), verbal (e.g., threats), relational (e.g., social exclusion) and indirect (e.g., spreading rumors) [3]. With the increased use of Internet and mobile phones, a new form of bullying has emerged, often labeled ‘cyber bullying’ [3]–[5]. In cyber bullying, aggression occurs via electronic forms of contact [6].

Increased exposure to the online environment has contributed to a heightened appreciation of the potential negative impact of cyber bullying [7]. Recent cross-sectional studies have shown an association between cyber bullying victimization and mental health problems, and even between cyber bullying victimization and suicide [4], [6], [8], [9]. Despite evidence from these cross-sectional studies, little is known with regard to the longitudinal impact of cyber bullying. To the best of our knowledge, only Schultze-Krumbholz et al. studied the longitudinal association between cyber bullying victimization and mental health problems in a relatively small sample (N = 233). They only showed a significant association between cyber bullying victimization and mental health problems in girls, not in boys [10].

The few available longitudinal studies examining the relationship between *traditional* bullying and mental health problems or suicide (ideation) show that being a victim of traditional bullying increases the risk of developing mental health problems and committing suicide later in life [6], [11]–[16]. However, longitudinal studies examining the associations between traditional bullying victimization and mental health problems or suicide (ideation) within large samples are still rare and further research is recommended [6].

Therefore, it is of interest to examine the longitudinal associations between traditional bullying and mental health and suicide (ideation), as well as the longitudinal associations between cyber bullying and mental health and suicide (ideation) in a large sample. The impact of traditional bullying victimization on mental health and suicide may be different than the impact ofcyber bullying victimization on mental health. It is possible that for example blocking online bullying messages, an option not available for face-to-face bullying, lessens the impact of cyber bullying on mental health while, in contrast, the possible breadth of audience on for instance websites may heighten the impact [3].

Furthermore, the impact of bullying victimization on boys may differ from the impact on girls. Few longitudinal studies have examined gender differences in victimization and mental health. These longitudinal studies indicate that both genders may have different risk profiles [6], [17]–[21], with girls who are victimized at baseline developing symptoms of depression or suicidal ideation at follow-up [10], [17], [20], [21] and boys not [10], [18]–[23].

The purpose of the current study was to examine whether traditional and cyber bullying victimization were associated with mental health problems and suicidal ideation at two-year follow-up (when controlling for mental health problems or suicidal ideation at baseline) in a large sample of adolescents. In line with previous findings [6], [17]–[23], we hypothesize that being a victim of *traditional* bullying is associated with mental health problems and suicidal ideation at two-year follow-up. In line with cross-sectional studies on cyber bullying victimization [4], [6], [8], [9], we hypothesize that *cyber* bullying victimization is associated with mental health problems and suicidal ideation at two year follow-up. Additionally, we explored whether bullying affects boys and girls in a different way, as previously suggested [6], [17]–[21].

## Methods

### Design and participants

A prospective study with two-year follow-up was conducted as part of the Rotterdam Youth Monitor (RYM), a longitudinal youth health surveillance system. The RYM monitors the general health, well-being, behavior and related factors of youth aged 0 to 19 years living in Rotterdam and the surrounding region in the Netherlands. The RYM is incorporated in the care (regular health examinations) of the preventive youth healthcare system; the RYM is used to detect (potential) individual health risks and problems in order to take the necessary preventive measures (including referrals for treatment).

The current study used RYM data from students at secondary schools. At baseline, the students were in their first year of secondary education (*M*_age_ = 12.50 years, *SD* = 0.62), and at follow-up in their third year (*M*_age_ = 14.31 years, *SD* = 0.58). Data were collected throughout the school year, except for July and August (Dutch summer holidays). The students completed a baseline questionnaire between September 2008 and July 2009 and a follow-up questionnaire between September 2010 and July 2011. Administration of the questionnaire took place at schools and was conducted by specially trained researchers and school nurses from the Municipal Public Health Service and/or by a teacher. In 2008–2009, 8,272 adolescents participated (95% participation rate), of whom 3,181 participated again in 2010–2011 (38%). The main reason for non-response (62%) at follow-up was schools being unwilling to participate again, which led to 49% of adolescents not being invited to participate at follow-up. Other reasons were: students were absent at the time of administering the follow-up questionnaire (about 5%), students had transferred to a school that did not participate at follow-up or students had repeated a school year (about 8%).

### Ethics statement

The data became available in the context of the government approved routine health examinations of the preventive youth health care. Separate informed consent was therefore not requested. Only anonymous data were used and the questionnaires were completed on a voluntary basis. Adolescents received verbal information about these questionnaires each time they were applied, whereas their parents received written information at every assessment point. Adolescents and their parents were free to refuse participation. Observational research with data does not fall within the ambit of the Dutch Act on research involving human subjects and does not require the approval of an ethics review board. As the data was provided anonymously tothe researchers, the study is not covered by the WMA Declaration of Helsinki.

### Measures

#### Bullying victimization

At baseline, two questions assessed whether the adolescent had been bullied in the past four week: 1) at school, and/or 2) via the Internet or via their telephone via Short Message Service (SMS). The response categories were: *Never, Once or twice, Once a week, Several times a week*, and *Daily*. For analysis purposes, being a victim of bullying at school (traditional victim), and being a victim on the Internet or via SMS (cyber victim) were dichotomized into the following categories: Never being victimized and Being victimized at least once or twice.

#### Mental health problems

At baseline and follow-up, mental health was assessed by the Dutch self-report version of the Strengths and Difficulties Questionnaire (SDQ) [24], [25]. The SDQ consists of 25 items describing positive and negative attributes of adolescents that can be divided into five subscales (five items each), i.e. emotional problems, conduct problems, hyperactivity-inattention, peer problems, and prosocial behavior. Each item is scored on a 3-point scale, with 0 =  ‘*not true*’, 1 =  ‘*somewhat true*’, and 2 =  ‘*certainly true*’. A total difficulties score is calculated by summing the scores on the emotional problems, conduct problems, hyperactivity-inattention and peer problems subscales (range 0–40; current study *α* = 0.74).

In line with other authors who divided their sample into subgroups (normal versus borderline/abnormal) based on questionnaire scores [13], [17], [19]–[23], we created two ‘mental health’ groups: normal (cut-off point SDQ total score at follow-up ≤80^th^ percentile; score ≤13) and borderline/abnormal mental health problems (cut-off point SDQ total score at follow-up >80^th^ percentile; score ≥14) [26]. These cut-off points were based on a large national survey in the Netherlands among 14–15 year-old adolescents [27].

#### Suicidal ideation

Suicidal ideation during the past 12 months was examined with one question at baseline and follow-up: ‘In the past 12 months, have you ever seriously considered ending your life?’. This item was scored on a 5-point scale: *Never, Once in a while, Sometimes, Often* and *Very often*. For analysis purposes, suicidal ideation was dichotomized in: Never had suicidal ideation over the last year; versus Had suicidal ideation at least once in a while over the last year.

#### Confounders

Age, gender, ethnicity, and level of education of the adolescent were measured at baseline and were incorporated as potential confounders in this study. Age was dichotomized into Below 13 years versus 13 years or older. Education was dichotomized into Basic or theoretical pre-vocational education versus, General secondary/pre-university education [28]. Ethnicity was classified as Dutch or non-Dutch in accordance with the definition of Statistics Netherlands [29]; i.e., adolescents with at least one parent born outside the Netherlands were classified as non-Dutch.

### Statistical analyses

All analyses were conducted using the total sample. Descriptive statistics were used to describe general characteristics of the study population. Differences in age, ethnicity, educational level, bullying victimization, mental health problems, and suicidal ideation between boys and girls were evaluated using chi-square tests. A chi-square test was also conducted to assess the association between traditional and cyber bullying victimization.

Furthermore, binary logistic regression analyses were used to assess the association between bullying victimization and mental health status or suicidal ideation at follow-up. Model 1 tested the association between traditional or cyber bullying victimization and mental health status or suicidal ideation at follow-up, adjusting for confounders (i.e., gender, age, ethnicity, and education) and the other type of bullying victimization. Model 2 also adjusted for baseline mental health status or suicidal ideation. Model 2 corresponds with the purpose of the study to examine the two-year longitudinal association between bullying victimization and mental health status or suicidal ideation, while controlling for mental health problems or suicidal ideation at baseline. In addition, we tested whether there were gender differences on mental health and suicidal ideation for the two types of bullying by respectively adding a Gender × Traditional bullying victimization (Model 3a) or a Gender × Cyber bullying victimization (Model 3b) interaction term to Model 2. If there was a significant Gender × Bullying victimization interaction, the results were described separately for boys and girls. Finally, we explored whether there were significant interactions between traditional and cyber bullying victimization on mental health and suicidal ideation. Odds ratios (OR) and their corresponding 95% confidence intervals (95% CI) were calculated.

Analyses were conducted using SPSS version 20. Results were considered significant at *p*<0.05, with the exception of interactions which were considered significant at *p*<0.10, in line with recommendations of Twisk [30].

## Results

### Non-response analysis

Differences between the boys/girls included in this study (*N* = 3181) and the boys/girls who did not participate in the follow-up assessment (*N* = 5091) were examined using chi-square tests (Table 1). Chi-square tests did not yield significant age differences between adolescents who participated at follow-up and who were lost-to-follow-up. However, group differences were found for education, ethnicity, mental health problems, suicidal ideation, and bullying victimization, with the lost–to-follow-up group having a lower education level, more often being of Dutch ethnicity, having more mental health problems, more suicidal ideation, and more often being a traditional and cyber bullying victim (only for girls) than the adolescents who participated at follow-up.

**Table 1:** Differences between boys/girls who did and did not participate at follow-up (N = 8271).

	Boys			Girls		
	Participated n = 1623 %	Lost-to-follow-up n = 2645 %	p value (χ^2^)	Participated n = 1558 %	Lost-to-follow-up n = 2445 %	p value (χ^2^)
**Age** (mean = 12.50, SD = 0.62)						
** **<13 years	53.9	51.2	0.09	58.6	56.0	0.09
**Ethnicity**						
** **Dutch	50.4	56.9	<0.001	46.3	55.4	<0.001
**Level of education**						
** **Basic or theoretical pre-vocational education	49.3	63.6	<0.001	51.0	64.2	<0.001
**Victim of bullying**						
** **Traditional alone	22.4	25.8	0.01	20.3	24.4	0.002
** **Cyber alone	4.7	5.3	0.45	5.5	9.0	<0.001
**Mental health problems**	20.5	24.9	0.001	20.5	25.5	<0.001
**Suicidal ideation**	13.8	17.5	0.002	23.9	26.8	0.04

### Descriptives

Mean age of adolescents in the current sample was 12.47 years (*SD* = 0.62); 51.0% of the sample consisted of boys and 48.4% was of Dutch ethnicity (Table 2). In total, 21.4% of the adolescents was a victim of traditional bullying and 5.1% was a victim of cyber bullying. No significant gender differences were found on bullying victimization (*p* = 0.10). Compared with boys, girls had significantly more mental health problems at follow-up (χ^2^ = 10.04; *p*<0.002) and suicidal ideation at baseline (χ^2^ = 52.42; *p*<0.001) and at follow-up (χ^2^ = 58.69; *p*<0.001). Furthermore, cyber bullying victims were more likely to also be traditional bullying victims compared to non-cyber bullying victims (boys: χ^2^ = 60.38; *p*<0.001; girls: χ^2^ = 29.21; *p*<0.001).

**Table 2:** General characteristics of the total study population, and by gender (N = 3181).

	Total	Boys	Girls	p value
	N = 3181	n = 1623	n = 1558	(χ^2^)
	%	%	%	
**Age** (mean = 12.47, SD = 0.62)				
** **<13 years	56.2	53.9	58.6	0.01
**Ethnicity**				
** **Dutch	48.4	50.4	46.3	0.02
**Level of education**				
** **Basic or theoretical pre-vocational education	50.1	49.3	51.0	0.33
**Victim of bullying**				0.10
** **Traditional alone	18.8	19.6	17.9	
** **Cyber alone	2.6	2.0	3.2	
** **Traditional and cyber	2.6	2.8	2.4	
**Mental health problems**				
** **At baseline	20.5	20.5	20.5	0.98
** **At follow-up	15.0	13.0	17.0	0.002
**Suicidal ideation**				
** **At baseline	18.8	13.8	23.9	<0.001
** **At follow-up	11.8	7.5	16.3	<0.001

### Bullying victimization and mental health problems

There was a significant interaction between gender and traditional bullying victimization (*p* = 0.08) (Model 3a) in the total sample (Table 3). Among boys, traditional bullying victimization was not significantly related to mental health problems in the fully-adjusted model (OR 1.03; 95% CI 0.72–1.47). Among girls, traditional bullying victimization was significantly related to mental health problems in the fully-adjusted model (OR 1.41; 95% CI 1.02–1.96).

**Table 3:** Associations of bullying victimization and mental health problems (N = 3181).

	Model 1		Model 2		Model 3a		Model 3b	
	OR (95%CI)	*p value*	OR (95%CI)	*p value*		*p value*		*p value*
**Sociodemographic characteristics**								
Gender, boy	0.73 (0.60–0.89)	0.002	0.71 (0.58–0.88)	0.001	0.80 (0.63–1.02)	0.07	0.76 (0.61–0.95)	0.01
Age, <13 years^a^	1.13 (0.92–1.39)	0.25	1.10 (0.89–1.38)	0.34	1.11 (0.90–1.38)	0.34	1.11 (0.90–1.39)	0.33
Ethnicity, Dutch	0.95 (0.77–1.17)	0.62	0.89 (0.72–1.10)	0.29	0.89 (0.72–1.11)	0.30	0.88 (0.71–1,09)	0.24
Education, basic or theoretical pre-vocational education	1.58 (1.27–1.96)	<0.001	1.23 (0.98–1.54)	0.08	1.23 (0.98–1.54)	0.08	1.23 (0.98–1.54)	0.08
**Bullying victimization**								
Traditional victim	1.64 (1.31–2.05)	<0.001	1.20 (0.95–1.53)	0.13	1.45 (1.06–2.00)	0.02	1.22 (0.96–1.54)	0.11
Cyber victim	2.35 (1.64–3.36)	<0.001	1.79 (1.23–2.61)	0.003	1.81 (1.24–2.65)	0.002	2.53 (1.55–4.12)	<0.001
**Mental health problems at baseline**			4.59 (3.68–5.73)	<0.001	4.59 (3.68–5.73)	<0.001		
**Gender × Traditional bullying victimization**					0.66 (0.42–1.54)	0.08		
**Gender × Cyber bullying victimization**							0.44 (0.20–0.95)	0.04

*Note*: OR  =  odds ratio; CI  =  confidence interval.

a Similar results were obtained when age was included as a continuous variable in the analysis.

Model 1 is adjusted for sociodemographic characteristics and bulling victimization. Mental health problems is the dependent variable.

Model 2 is the same as Model 1, but also adjusted for mental health problems at baseline.

Model 3a is the same as Model 2, but also includes a Gender × Traditional bullying victimization interaction term.

Model 3b is the same as Model 2, but also includes a Gender × Cyber bullying victimization interaction term.

There was a significant interaction between gender and cyber bullying victimization (*p* = 0.04) (Model 3b). Being a victim of cyber bullying was not related to mental health problems among boys (OR 1.18; 95% CI 0.64–2.17), whereas among girls, cyber bullying victimization was significantly related to mental health problems after controlling for baseline mental health (OR 2.38; 95% CI 1.45–3.91).

No significant interaction was found between traditional and cyber bullying victimization on mental health.

### Bullying victimization and suicidal ideation

No significant interaction was found between gender and traditional bullying victimization (*p* = 0.20) (Model 3a) and between gender and cyber bullying victimization (*p* = 0.48) (Model 3b) (Table 4). In the total sample, traditional bullying victimization was significantly related to suicidal ideation in the fully-adjusted model (Model 2: OR 1.56; 95% CI 1.21–2.02). Cyber bullying victimization was not associated with suicidal ideation after controlling for baseline suicidal ideation (Model 2: OR 1.22; 95% CI 0.80–1.87).

**Table 4:** Associations of bullying victimization and suicidal ideation (N = 3181).

	Model 1		Model 2		Model 3a		Model 3b	
	OR (95%CI)	*p value*	OR (95%CI)	*p value*		*p value*		*p value*
**Sociodemographic characteristics**								
Gender, boy	0.40 (0.32–0.51)	<0.001	0.48 (0.37–0.60)	<0.001	0.53 (0.40–0.70)	<0.001	0.49 (0.38–0.63)	<0.001
Age, <13 years^a^	0.89 (0.71–1.12)	0.31	0.90 (0.71–1.15)	0.39	0.90 (0.71–1.15)	0.39	0.90 (0.71–1.14)	0.39
Ethnicity, Dutch	1.06 (0.84–1.34)	0.63	1.10 (0.87–1.41)	0.42	1.11 (0.87–1.41)	0.41	1.10 (0.86–1.40)	0.44
Education, basic or theoretical pre-vocational education	1.32 (1.04–1.68)	0.02	1.17 (0.91–1.50)	0.22	1.17 (0.91–1.50)	0.22	1.17 (0.91–1.50)	0.22
**Bullying victimization**								
Traditional victim	1.95 (1.53–2.48)	<0.001	1.56 (1.21–2.02)	<0.001	1.77 (1.29–2.44)	<0.001	1.57 (1.21–2.03)	0.001
Cyber victim	1.74 (1.17–2.61)	0.007	1.22 (0.80–1.87)	0.36	1.23 (0.80–1.89)	0.34	1.36 (0.81 0 2.28)	0.24
**Suicidal ideation at baseline**			4.82 (3.79–6.12)	<0.001	4.84 (3.81–6.15)	<0.001	4.81 (3.79–6.10)	<0.001
**Gender × Traditional bullying victimization**					0.71 (0.43–1.20)	0.20		
**Gender × Cyber bullying victimization**							0.72 (0.29–1.79)	0.48

*Note*: OR  =  odds ratio; CI  =  confidence interval.

a Similar results were obtained when age was included as a continuous variable in the analysis.

Model 1 is adjusted for sociodemographic characteristics and bulling victimization. Suicidal ideation is the dependent variable.

Model 2 is the same as Model 1, but also adjusted for suicidal ideation at baseline.

Model 3a is the same as Model 2, but also includes a Gender × Traditional bullying victimization interaction term.

Model 3b is the same as Model 2, but also includes a Gender × Cyber bullying victimization interaction term.

A significant interaction was found between traditional and cyber bullying victimization on suicidal ideation (*p* = 0.01). Follow-up logistic regression analysis revealed that there was no further increased risk of developing suicidal ideation for adolescents being a victim of both types of bullying compared to adolescents being solely a victim of cyber (OR 1.35; 95% CI 0.86–2.12) or traditional bullying (OR 1.13; 95% CI 0.91–1.41).

## Discussion

This study shows that both *traditional* and *cyber* bullying victimization were associated with mental health problems in girls but not in boys, after controlling for baseline problems. Only *traditional* bullying victimization was associated with suicidal ideation after controlling for baseline suicidal ideation.

As hypothesized, but only among girls, traditional bullying victimization was associated with mental health problems after controlling for baseline mental health. This difference between boys and girls in the long-term effects of traditional bullying victimization on mental health is supported by various previous studies [6], [17]–[21]. The current study extends these findings to cyber bullying victimization, as we too found that the association between cyber bullying victimization and mental health problems was particularly driven by girls.

The gender differences in the impact of bullying on mental health found in our study may be partly explained by differences in the types of bullying (e.g. physical, relational) to which girls and boys are exposed. Regarding to traditional bullying, previous studies have found that girls more often experience relational victimization and that relational victimization has a greater impact on mental health problems than overt victimization, which is more often experienced by boys [31]–[33]. However, as the present study did not distinguish between different types of traditional or cyber bullying, it remains unclear whether the gender differences found in our study can be explained by the type of bullying. Therefore, future research should focus on different types of traditional bullying, as well as cyber bullying (e.g., via photos or video clips, emails), as different types of cyber bullying may also have different associations with mental health problems and suicidal ideation, and girls and boys may be exposed to different types of cyber bullying as well.

Furthermore, this study confirms the results of earlier studies indicating an association between traditional bullying victimization and suicidal ideation [6], [12], [15]. In contrast with our hypothesis, being a cyber bullying victim was not related to suicidal ideation after controlling for baseline suicidal ideation. A possible explanation for this discrepancy is the small size of the group of adolescents who were either a cyber bullying victim *and* had suicidal ideation. This may have resulted in limited power to detect a significant relationship between cyber bullying and suicidal ideation. Another possible explanation could be the difference in duration of exposure to the two types of bullying. Adolescents in our sample may have been exposed to cyber bullying for a shorter period of time compared to the time that they have been exposed to traditional bullying. This is in line with previous research showing that traditional bullying victimization remains relatively stable over time (between the ages of 8 and 16 years) [34], whereas cyber bullying victimization may occur at a later age, around the age of 14 years [4], when children spend more time on their mobile phones and are more likely to participate on social network sites (e.g. Facebook, MySpace) which are likely places for cyber bullying to occur [35]. It is possible that on the long-term, suicidal ideation only develops as a result of more pronounced and further developed mental health problems [36] and/or after persistent long-term exposure to bullying, as may have been the case with traditional bullying, but perhaps not yet with cyber bullying in our sample. Future research is required to gain more insight into these associations.

The purpose of the current study was to examine if bullying victimization was associated with mental health problems and suicidal ideation at follow-up. Nevertheless, analyzing the cross-sectional associations and the change in the percentage of adolescents with problems between baseline and follow-up among the different bullying victimization subgroups could provide additional information. Exploratory analyses on the baseline data (cross-sectional analyses) showed similar results as the longitudinal analyses described in the results section of this manuscript. As is often the case, our cross-sectional analyses yielded somewhat stronger associations between both types of bullying victimization and mental health and suicidal ideation than our longitudinal analyses. No significant interactions were found between gender and bullying victimization on mental health or suicidal ideation. This could indicate that the short term impact of bullying victimization on adolescents' mental health is similar for boys and girls, but that the long term impact of bullying on the mental health is different for boys and girls. Furthermore, additional analyses showed that the proportion of adolescents with mental health problems in the bullying victimization group significantly decreased more over the two year follow-up period compared to adolescents in the non-bullying victimization group (data not shown). However, it must be noted that percentage of mental health problems at two year follow-up was still higher in the bullying victimization group than in the non-bullying group. The same results were found for suicidal ideation. The only exception was that no significantly different change in the proportion of mental health problems in girls in the cyber bullying victimization group over the two-year follow-up period was found compared to girls who were not a cyber bullying victim at baseline.

The present study has both strengths and limitations that need to be addressed. A strength of the study is its longitudinal nature. The dataset provided the opportunity to explore relationships between the particular variables of interest within a large sample. Furthermore, many studies on cyber bullying are conducted online, and, therefore, may have a bias toward the experiences of adolescents who use the Internet more frequently. However, this study also has some limitations. First, not all adolescents in the study were available for analyses due to non-participation at follow-up. A non-response analysis showed that the adolescents who did not participate at follow-up had a lower educational level, were older, more often of Dutch ethnicity, more often a traditional or cyber bullying victim, and more often had mental health problems and suicidal ideation at baseline. Although we included these variables as confounders and adjusted for baseline problems in our analyses, it is possible that this selective drop out led to underestimation of the size of the association between bullying victimization and mental health problems or suicidal ideation, since a vulnerable group (i.e. a group with a high risk of mental health problems and suicidal ideation) dropped out. However, additional analyses showed that the relationship between both types of bullying victimization and mental health or suicidal ideation at baseline did not significantly differ between adolescents who dropped out and adolescents who did not drop out at follow-up. Nevertheless, the current findings should be generalized with caution, and we propose replication in large and varied populations. Second, traditional and cyber bullying victimization were assessed using single, self-reported items. Moreover, there is currently no consensus among researchers how to measure cyber bullying, and the changing nature of communication technology makes it difficult to establish a fixed definition. Third, mental health and suicidal ideation were also assessed using self-reported items, which may have resulted in less reliable outcomes. Nevertheless, research suggests that adolescents are better reporters of their own mental health status than parents and teachers [37].

In conclusion, our findings suggest that traditional bullying victimization is associated with an increased risk of suicidal ideation, and traditional and cyber bullying victimization are associated with an increased risk of mental health problems among girls. Future research should examine the mechanisms responsible for this differential response of girls and boys to the stress caused by bullying victimization. Furthermore, based on our results and results of other studies, studies on the current topic may want to consider differentiating between boys and girls. Our findings stress the importance of programs aimed at reducing bullying behavior in schools and online. These programs are particularly important because early-onset mental health problems may pose a risk for the development of psychiatric disorders in adulthood [38]–[40]. Moreover, although several intervention programs are available that reduce bullying behavior and victimization in schools [41], [42] such programs should not solely focus on school bullying. Prevention of cyber bullying should also be included in school anti-bullying policies [3], [4] since this is currently often lacking [43]. While some traditional methods for reducing bullying may be useful for cyber bullying too (e.g., peer support), more specific interventions will also be needed to reduce cyber bullying, such as how to contact mobile phone companies and internet service providers [3].
